# Engineering *Pichia pastoris* with surface-display minicellulosomes for carboxymethyl cellulose hydrolysis and ethanol production

**DOI:** 10.1186/s13068-020-01749-1

**Published:** 2020-06-15

**Authors:** Ce Dong, Jie Qiao, Xinping Wang, Wenli Sun, Lixia Chen, Shuntang Li, Ke Wu, Lixin Ma, Yi Liu

**Affiliations:** 1 State Key Laboratory of Biocatalysis and Enzyme Engineering, School of Life Sciences, Hubei University, Wuhan, 430062 Hubei China; 2 Hubei Key Laboratory of Industrial Biotechnology, School of Life Sciences, Hubei University, Wuhan, 430062 Hubei China; 3 BravoVax Co., Ltd., Wuhan, 430000 Hubei China

**Keywords:** Cellulosome, *Pichia pastoris*, Consolidate bioprocessing (CBP), Carboxymethyl cellulose (CMC), Bioethanol

## Abstract

**Backgrounds:**

Engineering yeast as a consolidated bioprocessing (CBP) microorganism by surface assembly of cellulosomes has been aggressively utilized for cellulosic ethanol production. However, most of the previous studies focused on *Saccharomyces cerevisiae*, achieving efficient conversion of phosphoric acid-swollen cellulose (PASC) or microcrystalline cellulose (Avicel) but not carboxymethyl cellulose (CMC) to ethanol, with an average titer below 2 g/L.

**Results:**

Harnessing an ultra-high-affinity IM7/CL7 protein pair, here we describe a method to engineer *Pichia pastoris* with minicellulosomes by in vitro assembly of three recombinant cellulases including an endoglucanase (EG), an exoglucanase (CBH) and a β-glucosidase (BGL), as well as a carbohydrate-binding module (CBM) on the cell surface. For the first time, the engineered yeasts enable efficient and direct conversion of CMC to bioethanol, observing an impressive ethanol titer of 5.1 g/L.

**Conclusions:**

The research promotes the application of *P. pastoris* as a CBP cell factory in cellulosic ethanol production and provides a promising platform for screening the cellulases from different species to construct surface-assembly celluosome.

## Background

Cellulosic biomass derived from low-value agricultural and wood pulping wastes is likely the most abundant renewable resource in the world [1–3]. In the past decades, production of bioethanol from cellulose has increasingly attracted attention due to the low cost and environmental friendliness. However, conversion of cellulose into fermentable sugars capable of utilization by microbes is still challenging [2, 4], largely limiting the industrial production of cellulosic bioethanol. In a traditional process, cellulosic biomass is degraded by commercial cellulases followed by microbial fermentation, leading to the production is time consuming and expensive [5–7]. To address the issue, several new strategies have been developed such as secretory expression of cellulases by bacteria [8], in vivo assembly of cellulosomes [9, 10] within microorganisms [11–13], as well as the consolidated bioprocessing (CBP) that combines enzyme production, cellulose hydrolysis, and biological fermentation into a single process [14, 15].

Yeast, especially for *Saccharomyces cerevisiae*, has been widely considered as an ideal CBP candidate for ethanol production due to its high ethanol productivity and strong ethanol tolerance [16]. In early studies, cellulases were cell-secreted in *S. cerevisiae* culture medium or independently displayed on the cell surface [17, 18], but the bioethanol yields were often quite low. Currently, the works demonstrated that in vivo or in vitro assembly of multiple cellulases on the *S. cerevisiae* cell surface in a structure termed cellulosome [19, 20] can significantly increase the ethanol yields [21–24]. In nature, the cellulosome is a complicated multi-enzyme machine produced by many cellulolytic microorganisms [25, 26]. Those methods required displaying multiple components on the yeast surface, including heterogeneous dockerin–cohesin pairs, carbohydrate-binding modules (CBMs) and appropriated bacterial cellulases, leading to low displaying efficiency sometimes. To date, microcrystalline cellulose (Avicel) or phosphoric acid-swollen cellulose (PASC) has been successfully utilized as the substrate for yeast fermentation, though the ethanol yields cannot meet the requirement of industrial production. Moreover, carboxymethyl cellulose (CMC) is difficult to be converted by *S. cerevisiae*, possibly because of its ultra-high viscosity that weakens the diffusion of hydrolysis products and influences the ethanol fermentation [27, 28]. Collectively, more effort is needed to achieve the goal of industrial production of cellulosic ethanol using yeast as the CBP cell factory.

**Fig. 1:**
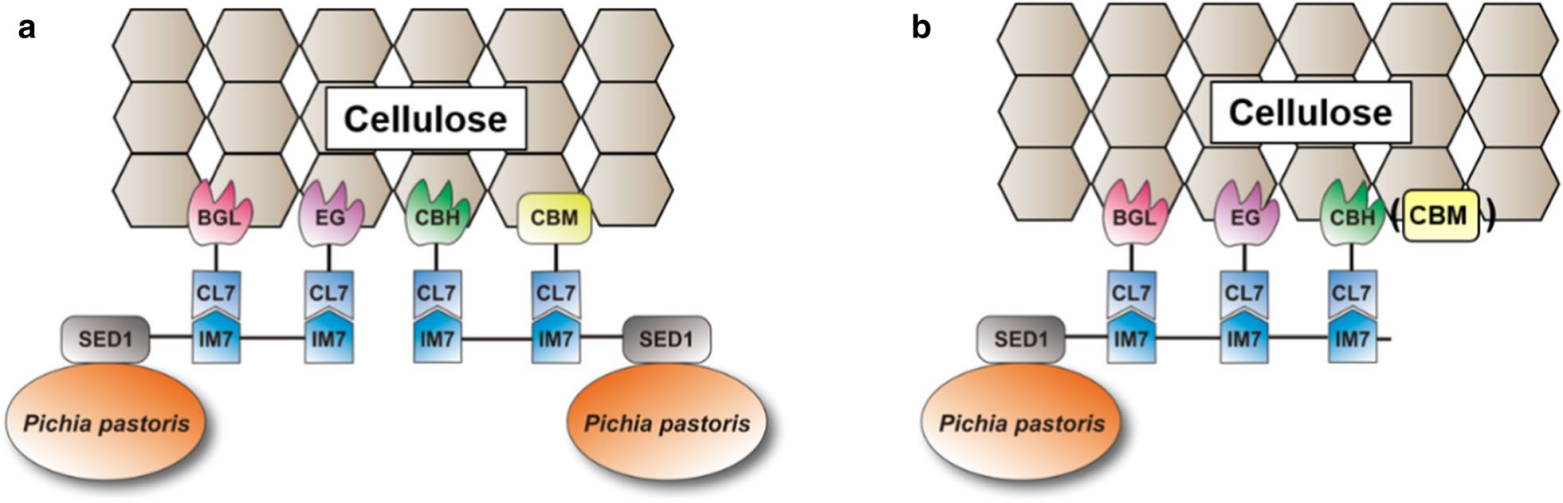
In vitro assembly of minicellulosomes on the *P. pastoris* cell surface. The ultra-high-affinity IM7/CL7 protein pair was used as the dockerin–cohesin pair for the yeast display system. The IM7 proteins were repeatedly displayed for (**a**) twice or (**b**) three times on the yeast cell surface. The three cellulases including an endoglucanase (EG), an exoglucanase (CBH) and a β-glucosidase (BGL), as well as a carbohydrate-binding module (CBM) were fused with an N-terminal CL7 tag and recombinantly expressed in *E. coli*

Recently, we developed an indirect *Pichia pastoris* surface-display method [29] that can simply display various enzymes with an average efficiency ten times higher than that of commonly used *S. cerevisiae* surface-display methods. Compared with *S. cerevisiae*, *P. pastoris* can achieve a much higher cell density in fermentation [1]. In practical applications, *P. pastoris* has been successfully applied in whole-cell biocatalysis for biodiesel production [30]. Therefore, we believe that *P. pastoris* is more suitable for catalyzing the reactions in greater viscosity, such as conversion of the high-viscosity CMC to ethanol. In this work, we want to expand our approach for construction of minicellulosomes on the *P. pastoris* cell surface, and then employ the engineered yeasts to produce cellulosic bioethanol. First, we harnessed an ultra-high-affinity IM7/CL7 protein pair [31] rather than the conventional dockerin–cohesin pairs for cellulosome assembly. In this system, the CL7 tag that engineered from the *E. coli* Colicin E7 DNase (CE7) retains the ultra-high-binding affinity (*K*_D_ ≈ 10^−14^–10^−17^ M) with its inhibitor Immunity protein 7 (IM7). According to the design (Fig. 1), the IM7 scaffoldin proteins were repeatedly displayed for twice or three times, generating Y-IM2 and Y-IM3 yeasts, respectively.

Diverse cellulases including an exo-mode cellobiohydrolase (CBH) from *Yarrowia lipolytica* [32], an endoglucanase (EG) from *Clostridium thermocellum* DSM1237 [33], a glucose-tolerant β-glucosidase (BGL) from *Thermoanaerobacterium thermosaccharolyticum* DSM 571 [34], and a CBM from *Thermobifida fusca* [35] were fused with N-terminal CL7 tags and recombinantly expressed in *Escherichia coli*. After that, the engineered *P. pastoris* yeasts were in vitro incubated with the *E. coli* lysates containing cellulases and CBM, leading to the assembly of minicellulosomes on cell surface. The cellulase activity assay indicated that Y-IM2 and Y-IM3 were able to hydrolyze microcrystalline cellulose (Avicel), phosphoric acid-swollen cellulose [PASC (86.2)] or carboxymethyl cellulose (CMC) to reducing sugars, with the enzyme activity comparable to or higher than free cellulases.

Finally, we employed the engineered yeasts as CBP cell factories to directly break down and ferment Avicel, PASC or CMC, producing ethanol with a titer of 2.5 g/L for Avicel and 1.2 g/L for PASC, respectively. Surprisingly, CMC is preferred for bioethanol fermentation, achieving an impressive ethanol titer of 5.1 g/L. To the best of our knowledge, this is the first time an engineered yeast can efficiently and directly transfer CMC to bioethanol. Moreover, the *P. pastoris* yeast with minicellulosomes can be lyophilized as the compound cellulases without loss of enzyme activity, showing great potential for industrial applications. Taken together, we develop a promising CBP platform for cellulose hydrolysis and bioethanol production by engineering the *P. pastoris* with surface-display minicellulosomes.

## Results

### Repeatedly displaying IM7 scaffoldins on the *P. pastoris* cell surface

**Fig. 2:**
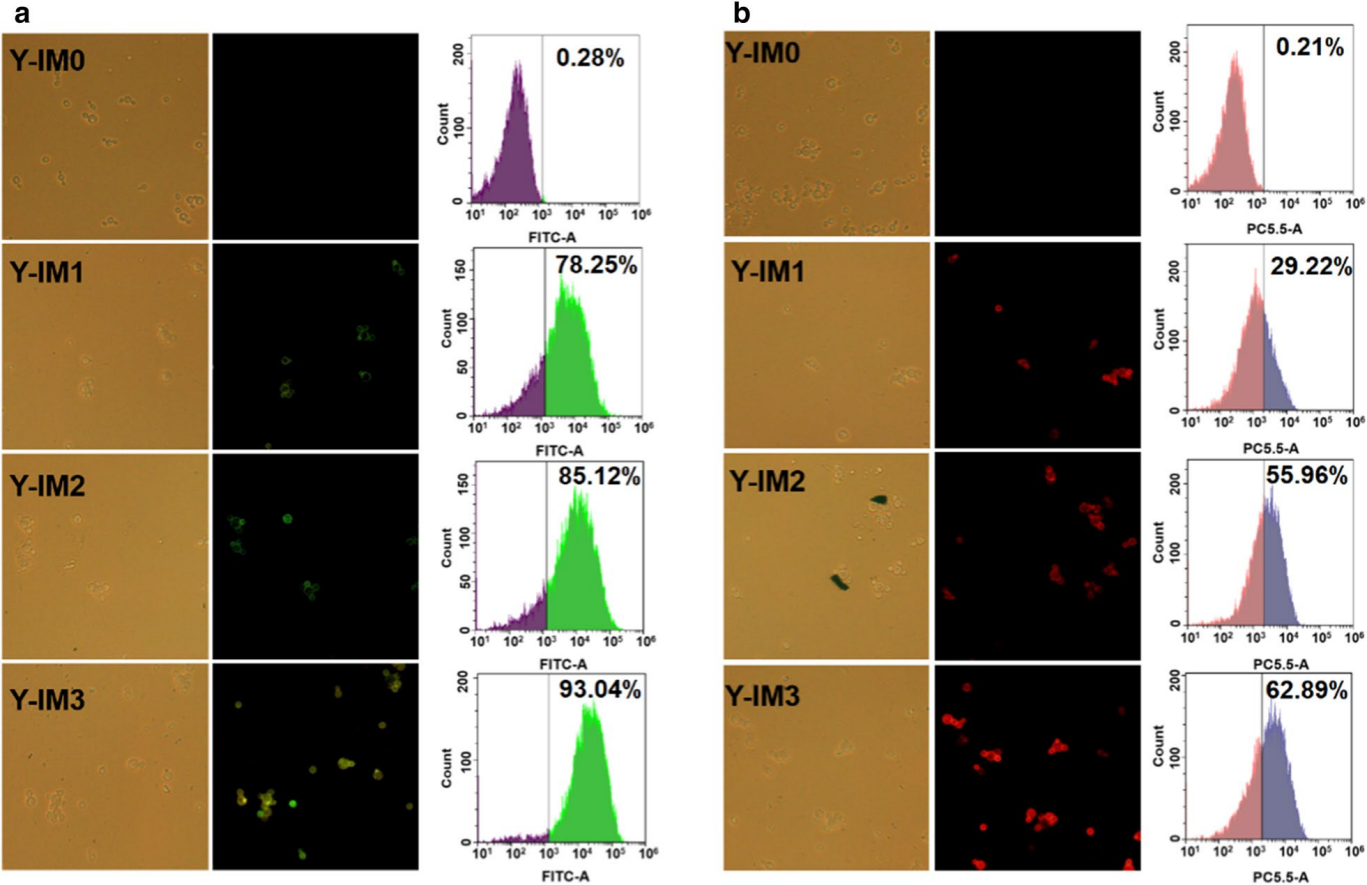
Fluorescence microscopy and flow cytometry analysis of scaffoldin surface display. All the yeast cells were treated with (**a**) mouse anti-HA tag monoclonal antibodies together with FITC (fluorescein isothiocyanate)-conjugated goat anti-mouse IgG antibodies, or with (**b**) CL7-mCheery red fluorescent proteins

In conventional yeast cell surface-display methods, the dockerin–cohesin pairs from bacterial cellulosomes are adopted, in which the dockerin is roughly a 10-kDa calcium-binding module that non-covalently associates with the scaffoldin (cohesin) at affinity in the sub-nM (~ 10^−6^ M) range [19]. In this work, the ultra-high-affinity IM7/CL7 protein pair (Fig. 1) was harnessed for cellulosomes’ assembly [31]. The 16 KDa CL7 is a catalytically inactive mutant of *E. coli* Colicin E7 (CE7) DNase with a pretty low *K*_D_ (~ 10^−14^–10^−17^ M) toward its binding 10 KDa partner immunity protein 7 (IM7). Based on the IM7/CL7 system, we recently developed an indirect *P. pastoris* surface-display method, achieving a tenfold increase in the display efficiency [29]. We believed that the ultra-strong protein–protein interaction between IM7 and CL7 would be helpful for cellulosome assembly. As shown in Fig. 1, the yeast surface anchor protein SED1 from *S. cerevisiae* without its signal sequence was fused to the IM7 scaffoldins. The surface localization of IM7 scaffoldins was confirmed by immunofluorescence microscopy and FACS (Fig. 2a). As a control, the wild-type Y-IM0 yeast without modification was not immunostained, whereas the Y-IM1, Y-IM2 and Y-IM3 variants were all in green color in the presence of mouse anti-HA monoclonal antibodies and FITC-conjugated goat anti-mouse antibodies. These results indicated that all engineered yeasts displayed the IM7 scaffoldins on the cell surface. As expected, the display efficiency was elevated with increasing the numbers of IM7. Furthermore, the engineered yeasts were all in red color in the presence of CL7 tagged mCherry fluorescent proteins (Fig. 2b). The FACS (Fig. 2b) confirmed that repeatedly anchoring the IM7 proteins can enhance the display efficiency. Compared with current yeast surface-display systems, in which less than 50% of cells were positively stained by immunofluorescence [21], over 90% positive cells were observed by our method. The huge increase in display efficiency was attributed to the use of ultra-high-affinity IM7/CL7 protein pair.

### In vitro assembly of minicelluolosomes on the *P. pastoris* cell surface

Previously, researchers assembled functional minicellulosomes in vitro on the *S. cerevisiae* yeast cell surface by incubation of the engineered yeast that has a chimeric scaffoldin [9, 10] or two miniscaffoldins [20, 21] with exogenous recombinant cellulases. There are two strategies for cellulosome assembly, in vitro or in vivo modes. Using the in vitro strategy, researchers obtained twofold higher bioethanol yields [12, 13, 23, 24], possibly due to the metabolic load was lowered in these yeast strains since they had no need to express and secrete the cellulases. Thus, here we also chose the in vitro mode to construct minicelluolosomes on the *P. pastoris* cell surface. Three different cellulases, including a CBH from *Yarrowia lipolytica*, an EG from *Clostridium thermocellum* DSM1237, a BGL from *Thermoanaerobacterium thermosaccharolyticum* DSM 571, as well as a CBM from *Thermobifida fusca*, were fused with N-terminal CL7 tags and recombinantly expressed and purified from *E. coli*. Notably, we had also tried to add the CL7 tag in the C-terminus, but some of cellulases were not expressed well. Based on our experiences, fusing the CL7 tag in the N-terminus can usually promote the solubility and production of the target proteins. Finally, the purified enzymes were incubated with Y-IM2 or Y-IM3 yeast.

**Fig. 3:**
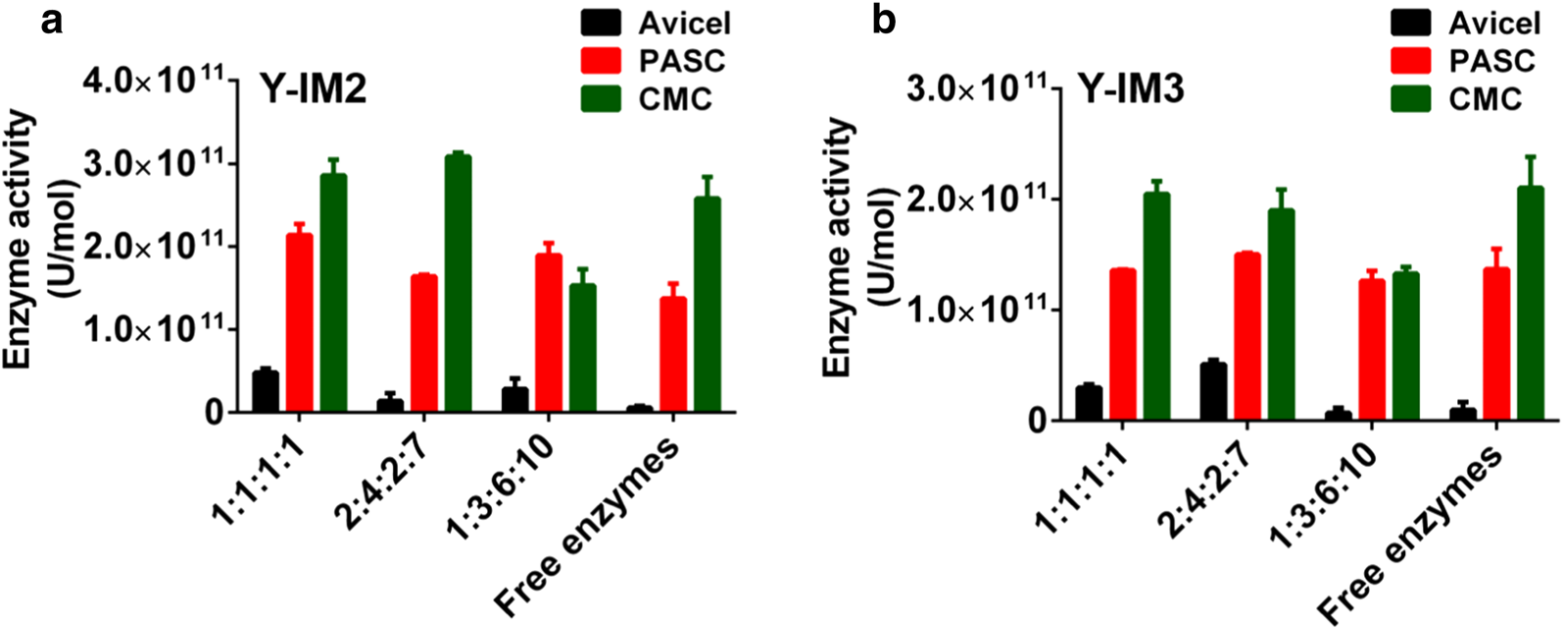
Enzyme activity of minicellulosomes on the cell surface of (**a**) Y-IM2 or (**b**) Y-IM3 yeast against Avicel, PASC and CMC at the different ratios of EG, CBH, BGL and CBM. The free cellulases were used as a control. It should be noted that this enzyme activity assay was detected by determination of the reducing sugars within the first 30 min

To prove construction of minicellulosomes, Avicel, PASC (86.2), or CMC was utilized as the substrate for enzyme activity assay according to the protocol [31]. This experiment was for preliminary screening the optimal ratio of each enzyme. We adjusted the ratio of EG, CBH, BGL and CBM at 1:1:1:1, 2:4:2:7 and 1:3:6:10, respectively. Meanwhile, the sample of free cellulases at 1:1:1:1 was used as the control [36]. The rational of the cellulases ratio is based on the previous reports [12, 37] as well as our own preliminary screening experiments. The data (Fig. 3) indicate that the enzyme activity of Y-IM2 and Y-IM3 is comparable to or higher than that of free cellulases. Importantly, we must point out that the enzyme activity shown in Fig. 3 was detected by determination of the reducing sugars within the first 30 min. Therefore, it does not equal to the hydrolysis capacity of yeast cells during the whole fermentation process, which had been investigated below. As expected, both minicellulosomes and the free cellulases showed higher activity toward CMC and PASC than Avicel (Fig. 3), though the improvement of the enzyme activity caused by minicellulosomes toward Avicel was more obvious (~ 2.6-fold). Based on these results, we chose 1:1:1:1 and 2:4:2:7 as the optimized ratios for the following fermentation experiments.

### Direct fermentation of celluloses to ethanol

**Fig. 4:**
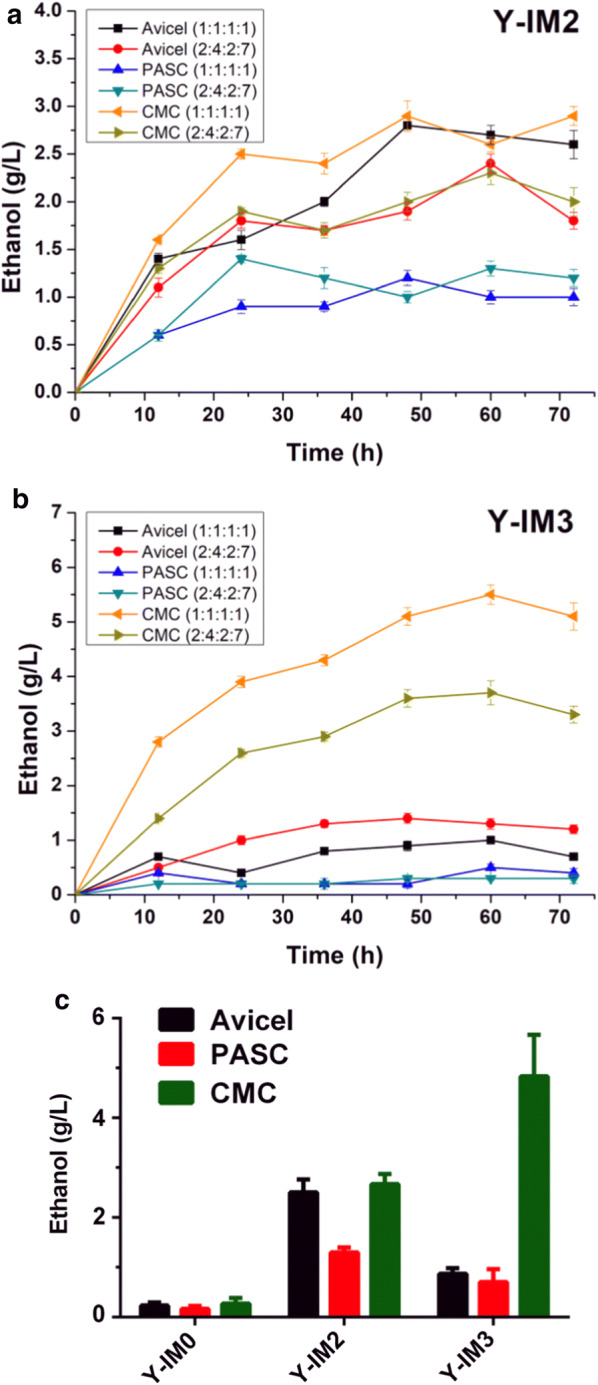
Direct ethanol production from three cellulose substrates using different yeast variants. Time profiles of the ethanol production from the yeast (**a**) Y-IM2 or (**b**) Y-IM3. The numbers in the bracket represented the ratios of EG, CBH, BGL and CBM used in the experiment. **c** The highest ethanol production of each cellulose substrates was shown. As the control, the wild-type Y-IM0 yeast was treated with *E. coli* lysate containing the enzymes

Direct ethanol fermentation from Avicel, PASC (86.2) or CMC was examined using the Y-IM2 and Y-IM3 yeast variants with *E. coli* lysate containing cellulases and CBM (Fig. 4a, b). The data demonstrated that the ethanol titer quickly increased within 60 h for all three substrates. Besides, Y-IM2 was better as the ethanol producer than Y-IM3 toward Avicel and PASC, achieving the highest ethanol yield at 2.5 g/L for Avicel and 1.2 g/L for PASC (Fig. 4c), respectively. In the previous studies of *S. cerevisiae*, Avicel or PASC was always the better substrate than CMC, yet the average ethanol production was lower than 2 g/L. Herein, employing our *P. pastoris* system obtained the same-level ethanol production when using Avicel or PASC as the substrate. Surprisingly, CMC was the best carbon source for both Y-IM2 and Y-IM3, achieving a highest ethanol yield of 5.1 g/L by Y-IM3 (Fig. 4c). As a control, the wild-type Y-IM0 yeast without engineering was used, which almost showed no ethanol production (less than 0.3 g/L) (Fig. 4c, left columns). This result indicated that the assembly of cellulosomes on Y-IM2 or Y-IM3 yeast cell surface was effective.

**Fig. 5:**
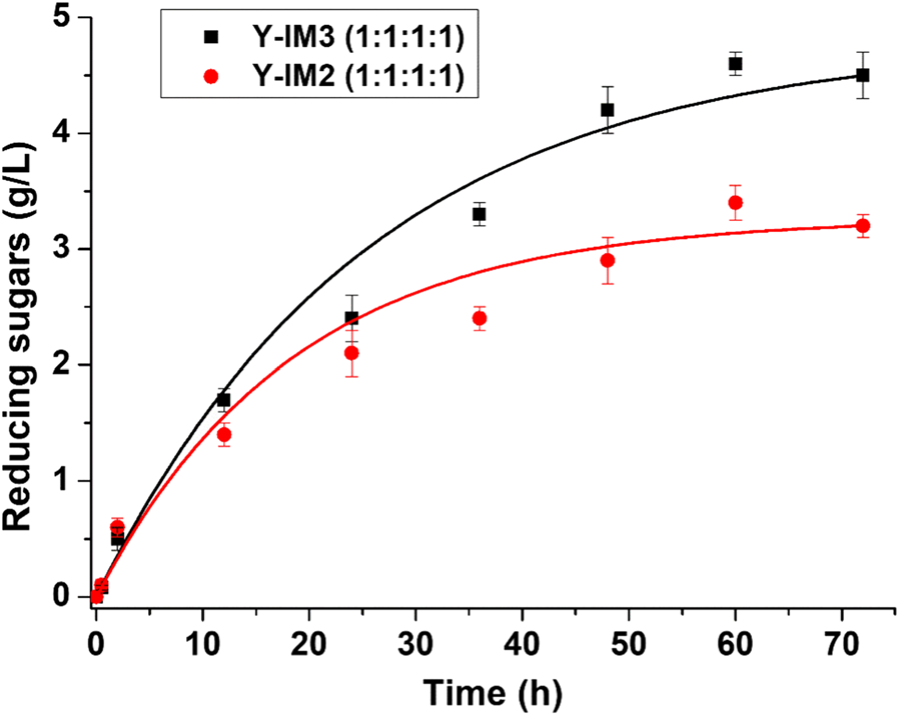
Whole-cell hydrolysis of CMC by the yeast Y-IM2 and Y-IM3 during the fermentation process

Moreover, we investigated the synergistic effect on CMC hydrolysis by examination of the reducing sugars produced in the fermentation. The data in Fig. 5 showed that the sugar concentration in Y-IM3 was higher than Y-IM2 after 12 h, indicating that more sugars were available for consumption by Y-IM3. In other words, the rate of CMC hydrolysis for Y-IM3 was higher than Y-IM2 during the whole fermentation process, confirming that the synergy effect increased with the number of IM7 scaffoldins being increased. These data are consistent with the ethanol production result (Fig. 4).

### Lyophilization of yeast cells as the compound cellulases

The commercial cellulase is often supplied as the compound of three types of cellulases including EG, CBH and BGL. The annual consumption of cellulases is very huge in many industrial fields [1], particularly in the breeding industry. As known to all, *P. pastoris* is considered as a GRAS (generally recognized as safe) microorganism by FDA and has been employed to produce diverse human peptides and proteins. In addition, *P. pastoris* has a strong cell wall and outer membrane capable of serving as a stable biomaterial for enzyme immobilization. Inspired by this knowledge, we believe that the *P. pastoris* with surface-display minicellulosomes might be directly used as the compound cellulases for industrial needs. To this end, we initially detected whether the dead yeasts with surface-display cellulosomes have the enzyme activity or not. The results (Additional file 1: Fig. S6) demonstrated that the catalytical activity was unchanged within 3 months when the yeast cells were stored at − 20 °C. Thereafter, the Y-IM2 and Y-IM3 with minicellulosomes were lyophilized for long-term storage. No loss of enzyme activity was observed when recovering the yeast cells in the buffer solution, proving that the lyophilized *P. pastoris* can be utilized as the compound cellulases. Most importantly, such kind of lyophilized *P. pastoris* with cellulosomes can be rapidly produced at large scale and low cost, showing great potential in industry.

## Discussion

Plenty of works have demonstrated that assembly of cellulosomes on the *S. cerevisiae* cell surface can enable it as a CBP cell factory to produce ethanol from cellulose. However, the application of such engineering strategy was limited largely due to the low display efficiency of cellulases and/or the high metabolic burden of the host yeast. To enhance the bioethanol production, several attempts have been made such as screening the dockerin–cohesin pairs [22], introducing the double-layered scaffoldins [23, 24], and adjusting the cellulase species and ratios [21, 37], etc. However, the minicellusomes in these works were constructed through hydrophobic interaction, hydrogen bond, or disulfide bond, leading to relatively low surface-display efficiency and poor stability. In addition, the engineering *S. cerevisiae* only produced less than 2 g/L ethanol from the conversion of Avicel or PASC. No obvious bioethanol was produced from CMC due to its ultra-high viscosity might cause problems in the fermentation.

In this study, to improve the display efficiency and availability of CMC, we describe a new strategy by engineering *P. pastoris* with tightly linked cell surface minicellulosomes. Compared with current yeast surface-display systems, the repeatedly anchoring IM7 scaffoldins on the *P. pastoris* cell surface demonstrated significantly enhanced display efficiencies, from ~ 50 to ~ 90%. The minicellulosomes were efficiently assembled by the introduction of an ultra-high-affinity IM7/CL7 protein pair [31]. Specially, four components including EG, CBHI, BGL and CBM from distinct bacteria were fused with N-terminal CL7 tags, purified from *E. coli*, and in vitro incubated with Y-IM2 or Y-IM3 variant at different ratios, resulting in comparable or better catalytic activity than that of free enzymes. We further investigated the synergy effect on CMC hydrolysis by determination of the reducing sugars produced by Y-IM2 and Y-IM3 in the fermentation, showing that more sugars were available for consumption by Y-IM3 than Y-IM2. This result confirmed that the synergy effect was enhanced by increasing the number of IM7 scaffoldins. Moreover, the engineered *P. pastoris* strains were lyophilized for long-term storage without cellulase activity losses, showing great potential as the compound cellulases instead of commercial cellulases in industry.

At last, we harnessed the engineered *P. pastoris* with minicellulosomes as the CBP cell factory to directly convert Avicel, PASC and CMC to ethanol. The highest ethanol yield was 2.5 g/L for Avicel and 1.2 g/L for PASC, which were comparable to or higher than those obtained by *S. cerevisiae* previously. More importantly, these results indicated that CMC was preferred for bioethanol fermentation in our *P. pastoris* system, achieving a highest titer of 5.1 g/L. To the best of our knowledge, it is the first successful work that realized efficient production of ethanol from CMC by yeast. Compared with the commonly used bioethanol producer *S. cerevisiae*, *P. pastoris* has been able to reach a much higher cell density in fermentation. It is therefore suitable for catalyzing the high-viscosity cellulose substrate such as CMC, which was consistent with what we have observed.

In this work, we found that optimizing the ratio of various cellulases would affect the cellulosome assembly and the ethanol production quite a lot. In future, higher cellulosic ethanol production might be achieved by further combinatorial optimization of the cellulase species and ratios.

## Conclusion

Taking advantage of the ultra-high-affinity IM7/CL7 system, we develop an efficient method capable of in vitro assembling minicellulosomes on the *P. pastoris* cell surface. The engineered yeasts with cellulosomes can be cost-effectively produced at large scale and lyophilized as the compound cellulases, showing great potential in industrial applications. For the first time, the engineered *P. pastoris* enabled efficient production of ethanol from direct conversion of CMC, achieving an impressive ethanol titer of 5.1 g/L. Collectively, the research promotes the application of *P. pastoris* as a CBP cell factory in cellulosic ethanol production.

## Methods

### Strains and media

*E. coli* DH5α was used as the host for DNA manipulations, and *E. coli* BL21(DE3) was the host for recombinant expression of CL7 tagged cellulases or CBM domains. *P. pastoris* strain GS115 and the vector pPICZαA were obtained from Invitrogen (Carlsbad, CA, USA). The vectors pET23a-T, pET23a-CL7, and pCDNA3.1-mCherry were constructed and stored in our laboratory previously [24]. The genes encoding exo-mode cellobiohydrolases (CBH) from *Yarrowia lipolytica*, endoglucanases (EG) from *Clostridium thermocellum* DSM1237, glucose-tolerant β-glucosidase (BGL) from *Thermoanaerobacterium thermosaccharolyticum* DSM 571, as well as CBM from *Thermobifida fusca* were synthesized by Sangon Biotech (Shanghai, China). The gene sequences are shown in the Additional file 1. *E. coli* strains were grown in LB medium (1% tryptone, 0.5% yeast extract, 1% NaCl) supplied with 100 μg/ml ampicillin. *P. pastoris* yeasts were grown first in YPD plates (1% yeast extract, 2% peptide, and 2% glucose) supplemented with 100 µg/mL of Zeocin, and then cultured in BMGY/BMMY medium base (20.0 g/L peptone, 10.0 g/L yeast extract, 100 mmol PBS broth, pH 6.0).

### Construction of plasmids

The plasmids pET23a-CL7-EG, pET23a-CL7-CBHI, pET23a-CL7-BGL, and pET23a-CL7-CBM were constructed by insertion of the corresponding genes into pET23a(+) vectors (Invitrogen, USA). The plasmid pPICZαA-HA-Im7-SED1 that produces *P. pastoris* Y-IM1 was described in the previous work [29]. The plasmids of Y-IM2 and Y-IM3 were constructed based on Y-IM1 plasmid by repeatedly inserting IM7 for twice or three times, namely pPICZαA-HA-2XIm7-SED1 and pPICZαA-HA-3XIm7-SED1, respectively. In addition, a “GGGGS”_2_ liker was added between each IM7 units. All the *P. pastoris* yeast plasmids were digested with *Pme1* and transformed into yeast cells to integrate the target genes into the yeast chromosome. The detailed protocol, schemes (Additional file 1: Figs. S1–S5), and primer pairs (Additional file 1: Table S1) are given in Additional file 1.

### Yeast surface-display and *E. coli* expression

All the *P. pastoris* yeast plasmids were digested with *Pme1* and transformed into GS115 competent cells. Transformants were first isolated by incubation at 28 °C for 48 h on YPD plates supplemented with 100 µg/mL of Zeocin. Then, five to ten single colonies of transformants were inoculated in 20 mL of BMGY in 250-mL flasks and cultivated at 28 °C under 200 rpm. After 24 h, the cells were centrifuged at 5000×g for 5 min, resuspended in 20 mL of BMMY medium containing 1% (v/v) methanol and continued to grow at 28 °C, 200 rpm for 24 h. To express CL7 tagged proteins in *E. coli*, 0.5 mM isopropyl-β-d-thiogalactopyranoside (IPTG) was added to the cells when the cells were grown to an OD_600_ of 0.6. Then, the cells were grown at 18 °C for 12 h. The *E. coli* cells collected were resuspended in PBS buffer containing 200 mM NaCl and 10 mM CaCl_2_ (pH 7.4) and then sonicated on ice for 20 min. The cell lysates were either purified by Ni–NTA affinity columns, or directly incubated with the engineered yeast strains in fermentation experiments.

### Fluorescence microscopy and flow cytometric analysis

The yeast strains including Y-IM0, Y-IM1, Y-IM2, and Y-IM3 were harvested and washed twice by ice-cold water, resuspended and blocked in 1 mL PBS buffer (200 mM NaCl, pH 7.4) with 1 mg/mL BSA for 1 h at 4 °C with rotation. Then, 1 µL of mouse anti-HA tag monoclonal antibodies or 5 µg of CL7–mCherry fluorescent proteins were added to the cell suspension of 1000 µL and then incubated at room temperature with rotation for 2 h. In the next, the cells were washed three times with PBS and resuspended in 200 µL of PBS with the addition of 1 µL of FITC-conjugated goat anti-mouse IgG(H + L) antibodies, followed by incubation of them at room temperature for 1 h with rotation. Finally, the cells were washed three times with PBS, resuspended in 1 mL of PBS and examined by a fluorescence microscopy (IX73, Olympus, Tokyo, Japan). Flow cytometric analysis (FACS) was analyzed with a flow cytometer (CytoFLEX, Beckman Coulter, Suzhou, China) to estimate the percentage of the fluorescence positive yeast cells.

### In vitro assembly of minicellulosome and enzyme activity assay

The induced Y-IM2 and Y-IM3 strains were mixed with the purified recombinant CL7 tagged enzymes in 100 mM Tris–HCl buffer with 10 mM CaCl_2_ (pH 8.0) at various ratios, and kept for 2 h at 4 °C for minicellulosome assembly [23, 24]. The enzyme activity of cellulosome or free cellulases against Avicel or PASC or CMC was detected by 3, 5-dinitrosaloculoc acid (DNS) assay [36]. The PASC (86.2) was prepared from Avicel (Sangon Biotech, Shanghai, China) as described previously [38]. Minicellulosomes or free cellulases were incubated with 0.1% cellulose substrate in 50 mM citrate buffer (pH 4.8) with 10 mM CaCl_2_ at 50 °C for 30 min. After addition of DNS and boiling for 10 min, the reducing sugars were detected at 540 nm. One unit of the enzyme activity was defined as the amount of enzyme that released 1 mol of product from the cellulose substrate at 50 °C in 1 min.

### Fermentation

After induction, the Y-IM0, Y-IM2 and Y-IM3 strains were washed twice with YP medium (1% yeast extract, 2% peptone, 10 mM CaCl_2_). Then, they were incubated with *E. coli* lysates containing enzymes at various ratios in the same buffer, and kept for 4 h at 4 °C to allow cellulosome assembly. Next, yeast cells with minicellulosomes were cultivated in YP medium with 1% cellulose substrate (Avicel, PASC, or CMC) to an OD_600_ of 50. Fermentation was performed anaerobically 100-mL flask at 30 °C with agitation at 250 rpm. The ethanol concentration was analyzed by an ethanol biosensor M-100 (Shellman Life Science, Shenzhen, China) supplied with polyaniline film-immobilized ***alcohol oxidase, which had been proven and in good agreement with the results of standard method obtained by gas chromatography [39]. Meanwhile, the reducing sugar concentration was determined by the biosensor instrument.

### Lyophilization of the yeast cells

The freshly induced YM-2 and YM-3 yeast cells were incubated with *E. coli* lysates containing EG, CBH, BGL and CBM at 1:1:1:1 in 100 mM Tris–HCl buffer with 10 mM CaCl_2_ (pH 8.0), and kept for 2 h at room temperature. The above mixtures were centrifuged for 10 min at 8000 rpm. After that, the cell pellets were collected and lyophilized at − 70 °C under vacuum using a LABCONCO freeze drier (Kansas City, Missouri, USA).

## Supplementary information

**Additional file 1.** Additional figures and table.

## Data Availability

All data generated or analyzed during this study are included in this published article and its additional files.

## Abbreviations

PASC: Acid-swollen cellulose
Avicel: Microcrystalline cellulose
CMC: Carboxymethyl cellulose (CMC)
CBP: Consolidated bioprocessing
CBM: Carbohydrate-binding module
CBH: Exo-mode cellobiohydrolases
EG: Endoglucanases
BGL: Glucose-tolerant β-glucosidase
FACS: Flow cytometric analysis
