# Supplementary material for: Engineering Pichia pastoris with surface-display minicellulosomes for carboxymethyl cellulose hydrolysis and ethanol production
Source: Biotechnol Biofuels. 2020 Jun 15;13:108. doi: 10.1186/s13068-020-01749-1 (PMC7296672; doi:10.1186/s13068-020-01749-1)
Supplement: Supplementary file 1 — Additional file 1. Additional figures and table. [file 13068_2020_1749_MOESM1_ESM.docx]

**Additional file**

**The gene sequences used in this work**

**CEL9D:**

ATGGCCAAAATCACCGAAAATTATCAGTTCGATTCTCGTATTCGCTTGAATAGCATTGGCTTTATTCCGAATCATTCTAAGAAAGCCACCATTGCCGCTAATTGTAGCACCTTTTATGTTGTCAAAGAAGATGGCACCATTGTTTATACCGGTACCGCCACCAGCATGTTCGATAATGATACCAAAGAAACCGTTTATATCGCTGATTTCTCTAGCGTCAATGAAGAAGGTACCTATTATTTGGCCGTTCCGGGCGTCGGTAAATCTGTTAATTTCAAAATCGCTATGAATGTCTATGAAGATGCTTTCAAAACCGCCATGTTGGGCATGTATTTGTTGCGTTGTGGTACCTCTGTTAGCGCCACCTATAATGGCATTCATTATTCTCATGGTCCTTGCCATACCAATGATGCTTATTTGGATTATATCAATGGCCAACATACCAAGAAAGATAGCACCAAAGGCTGGCATGATGCTGGTGATTATAATAAATATGTTGTCAATGCCGGCATTACCGTTGGTTCTATGTTTTTGGCTTGGGAACATTTTAAAGATCAGTTGGAACCGGTCGCCTTGGAAATCCCTGAAAAGAATAATAGCATCCCGGATTTTCTTGATGAATTGAAATATGAAATCGATTGGATTTTGACCATGCAATATCCTGATGGCTCTGGTCGTGTTGCCCATAAAGTCAGCACCCGCAATTTTGGTGGCTTTATTATGCCGGAAAATGAACATGATGAACGTTTCTTTGTTCCTTGGTCTAGCGCCGCTACCGCCGATTTTGTCGCTATGACAGCCATGGCCGCTCGTATTTTTCGCCCGTATGATCCTCAGTATGCTGAAAAATGTATTAATGCCGCTAAAGTTAGCTATGAATTTTTGAAAAATAATCCTGCTAATGTCTTTGCCAATCAATCTGGCTTTAGCACCGGTGAATATGCTACCGTTTCTGATGCCGATGATCGCTTGTGGGCCGCTGCCGAAATGTGGGAAACCTTGGGTGATGAAGAATATTTGCGTGATTTCGAAAATCGCGCTGCCCAGTTCTCTAAGAAAATTGAAGCTGATTTCGATTGGGATAATGTTGCCAATTTGGGCATGTTTACCTATTTGTTGTCTGAACGTCCGGGTAAAAATCCTGCCTTGGTTCAGAGCATTAAAGATTCTTTGTTGAGCACCGCTGATTCTATTGTCCGTACCAGCCAAAATCATGGCTATGGTCGCACCTTGGGCACCACCTATTATTGGGGCTGCAATGGTACCGTTGTCCGCCAGACCATGATTTTGCAAGTTGCCAATAAAATCAGCCCGAATAATGATTATGTTAATGCTGCCTTGGATGCTATCTCTCATGTCTTCGGTCGTAATTATTATAATCGCAGCTATGTCACCGGCTTGGGTATTAATCCGCCTATGAATCCTCATGATCGTCGCTCTGGCGCCGATGGTATTTGGGAACCGTGGCCTGGCTATTTGGTTGGTGGCGGTTGGCCGGGTCCTAAAGATTGGGTCGATATTCAGGATAGCTATCAAACCAATGAAATTGCCATTAATTGGAATGCTGCCTTGATTTATGCTTTGGCCGGCTTCGTTAATTATAATTCTGCTCAAAATGAAGTTTTGTATGGCGATGTCAATGATGATGGTAAAGTCAATTCTACCGATTTGACCTTGTTGAAACGCTATGTTTTGAAAGCCGTCAGCACCTTGCCGTCTAGCAAAGCTGAAAAGAATGCTGATGTTAATCGTGATGGTCGCGTTAATTCTAGCGATGTCACCATTTTGTCTCGTTATTTGATTCGCGTCATTGAAAAATTGCCTATTTAA

**CBHI:**

ATGCAGCAAGCCGGTACCGCCACCGCTGAAAATCATCCGCCTTTGACCTGGCAGGAATGTACCGCTCCGGGTTCTTGCACCACCCAAAATGGCGCTGTTGTCTTGGATGCCAATTGGCGTTGGGTTCATGATGTCAATGGTTATACCAATTGTTATACCGGCAATACCTGGGATCCGACCTATTGCCCTGATGATGAAACCTGTGCCCAGAATTGCGCTTTGGATGGTGCCGATTATGAAGGTACCTATGGCGTTACCTCTAGCGGCTCTAGCTTGAAATTGAATTTCGTTACCGGTTCTAATGTCGGCAGCCGTTTGTATTTGTTGCAGGATGATTCTACCTATCAAATCTTCAAATTGTTGAATCGCGAATTTTCTTTCGATGTTGATGTCAGCAATTTGCCGTGTGGTTTGAATGGCGCCTTGTATTTTGTTGCCATGGATGCTGATGGTGGCGTCTCTAAATATCCTAATAATAAAGCCGGTGCTAAATATGGTACCGGCTATTGTGATAGCCAGTGCCCGCGTGATTTGAAATTCATCGATGGTGAAGCTAATGTCGAAGGCTGGCAACCTTCTAGCAATAATGCCAATACCGGTATTGGCGATCATGGCTCTTGTTGCGCTGAAATGGATGTTTGGGAAGCTAATTCTATTAGCAATGCCGTCACCCCGCATCCTTGCGATACCCCTGGTCAGACCATGTGTTCTGGCGATGATTGCGGTGGCACCTATAGCAATGATCGTTATGCCGGTACCTGTGATCCGGATGGCTGCGATTTTAATCCTTATCGCATGGGCAATACCAGCTTTTATGGTCCGGGCAAAATTATTGATACCACCAAACCTTTTACCGTTGTCACCCAATTTTTGACCGATGATGGTACCGATACCGGCACCTTGTCTGAAATCAAACGCTTCTATATCCAGAATAGCAATGTTATTCCGCAACCTAATTCTGATATTAGCGGTGTCACCGGCAATTCTATTACCACCGAATTTTGTACCGCTCAGAAACAAGCCTTTGGTGATACCGATGATTTTAGCCAGCATGGTGGCTTGGCTAAAATGGGTGCCGCTATGCAGCAAGGCATGGTTTTGGTCATGTCTTTGTGGGATGATTATGCCGCTCAAATGTTGTGGTTGGATAGCGATTATCCGACCGATGCTGATCCGACCACCCCTGGTATTGCTCGCGGTACCTGCCCTACGGATTCTGGTGTTCCTAGCGATGTCGAATCTCAAAGCCCTAATTCTTATGTTACCTATAGCAATATCAAATTCGGTCCTATCAATTCTACCTTTACCGCCAGCAATCCGCCTGGTGGCAATCGTGGTACCACGACGACCCGTCGCCCTGCTACGACGACGGGTTCTAGCCCTGGTCCTACCCAGTCTCATTATGGTCAATGTGGTGGCATTGGTTATTCTGGTCCTACGGTTTGTGCTTCTGGTACCACCTGCCAGGTCTTGAATCCTTATTATAGCCAATGCTTGTAA

**BGL:**

ATGTCTGATTTCAATAAAGATTTTCTTTTCGGTGTTGCTACCGCCAGCTATCAGGTCGAAGGCGCCTATAATGAAGATGGTCGTTCTATGAGCATTTGGGATACCTTTTGTCGCCAAGATGGTAAAGTTTATAAAGGTCATAATGGCGATGTCGCTTGCGATCATTATCATTTGTATAAAGATGATGTTAAAATGATGAAAGATTTGGGCATTGAAGCTTATCGTTTTTCTATTGCCTGGCCGCGCATTTTTCCTGAAAAAGGCCATTATAATCCGAAAGGTATCGATTTCTATAAACGTTTGACCGATGAATTGTTGAAAAATGATATCAAACCGTTTGTTACCATTTATCATTGGGATTTGCCTCAGTGGGCCGATGATTTGGGTGGCTGGTTGAATCGTGAAGTTGTCGATTGGTTCGGTGAATATGTCAGCAAATTGTTTAATGAATTGGGTGGCTATATCCGCAATTGGATTACCTTGAATGAACCGTGGTGTTCTAGCTTTTTGTCTTATTTCATCGGTGAACATGCTCCTGGCCATAAAGATTTGGGTGAAGCCGTTTTGGTCAGCCATAATTTGTTGTTGGCTCATGGTAAAGCCGTTGAAATCTTCCGCGATATCAATTCTAGCGATTCTAAAATCGGCATCACCTTGAATTTGAATGAAGTCTTTCCGGCTACCGATAGCCCTGAAGATAAAGCCGCTGCCCGTATTGCCGATGGCTTTCAAAATCGCTGGTTTTTGGATCCGATCTTCAAAGGCGAATATCCTAAAGATATGTTGGAATTGTTTGGTAAATATGCTAAAACCGATTTCATCACCGATGGCGATTTGAAACGTATCTCTCAGAAATTGGATTTTCTTGGTGTTAATTATTATACCCGCGCTGTTGTTAAGAAAGGTAATGATGGCATTTTGAATGCCGAACAAATCGATGTTGATAATGAAAAAACCGAAATGGGCTGGGAAGTCTATCCGGAATCTTTGTATAATATCTTGATGCGTTTGAAAAATGAATATACCTTCGATTTGCCTTTGTATATCACCGAAAATGGTGCTGCCTATAAAGATGTTGTCAGCGATGATGGCCATGTTCATGATGAAAAACGCGTCGAATTTTTGAAAAAACATTTCAAACAGGCTAAACGTTTTATTGATGATGGTGGCAATTTGCGCGGCTATTTCGTCTGGTCTTTGATGGATAATTTCGAATGGGCCCATGGCTATAGCAAACGTTTCGGTATCGTTTATGTCGATTATGAAACCGAAAAACGCATTTTGAAAGATTCTGCTTTGTGGTATAAAAATTTGATCAGCACCCGTACCATTTAA

**CBM:**

ATGGGTGACGCTCCGGCTCTGGCTTGCGAAATCGAATACTCTACCCCGAACACCTGGTACGGTGGTTTCACCGCTCAGGTTCGTATCTACAACGGTTCTCACGAATCTATCGACGGTTGGGAACTGACCTGGGACTTCACCAACGGTGAAACCATCAACCAGGCTTGGAACGCTACCGCTCAGCAGTCTGGTACCACCGTTACCGTTACCAACGTTTCTTGGAACTCTACCATCCCGCACCACGGTTCTGTTGAATTCGGTTTCAACGCTAACTCTACCCGTGAACCGGGTGTTCCGGAAAACTTCAAACTGAACGGTTCTCTGTGCTCTGTTGCTTAA

**Construction of the *P. pastoris* surface display plasmids**

The plasmids for *P. pastoris* surface display of SED1 fusion proteins with a N-terminal HA tag were constructed as follows. The gene encoding SED1 protein without original secretion signal sequence was obtained by PCR from the genome of *S. cerevisiae* strain INVSC1 using the primers SED1F/SED1R. The above PCR products were used as templates for obtaining gene encoding HA-SED1 using the primers ZαHAF, HA-MCSF, MCS-GSF and SED1R. The restriction enzyme sites including *Nhe* I, *Nde* I, *Hpa* I, *Apa* I and a glycine-serine peptide linker were inserted between HA and SED1. The corresponding DNA fragment was cloned into the vector pPICZαA between *EcoR* I and *Sac* II, producing the plasmid termed pPICZαA-HA-SED1 (Fig S1). The *E. coli* Im7 gene was obtained by PCR from pPICZαA-Im7 using the primers Im7F and Im7R. The corresponding DNA fragment was cloned into pPICZαA-SED1 between *Nhe* I and *Apa* I, producing the plasmid called pPICZαA-HA-Im7-SED1 (Fig S2), which represents the Y-IM1 variant. The plasmid termed pPICZαA-HA-2xIm7-SED1, representing the Y-IM2 variant, was constructed following the protocol shown in Fig S3. The plasmid termed pPICZαA-HA-3xIm7-SED1, representing the Y-IM3 variant, was constructed following the protocol shown in Fig S4. Information regarding these yeast plasmids were illustrated in Fig S5. All the primers used are shown in Tab S1.


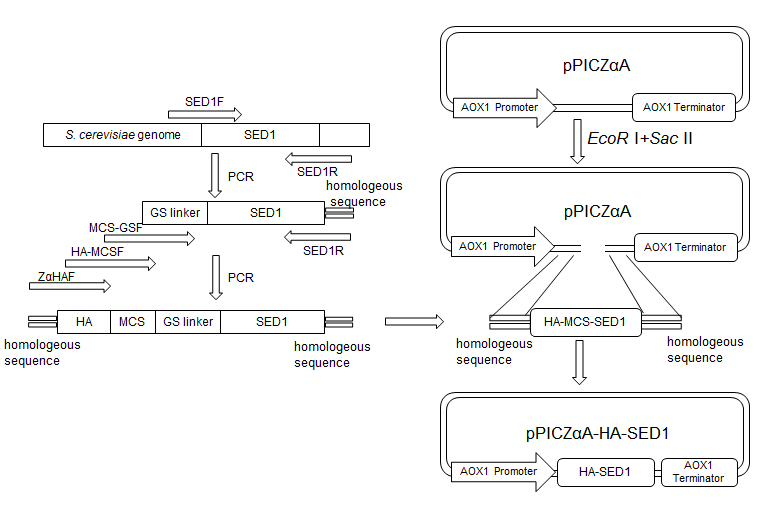


**Fig S1.** Scheme for the construction of plasmid pPICZαA-HA-SED1.

**Fig S2.** Scheme for the construction of plasmid pPICZαA-HA-Im7-SED1.
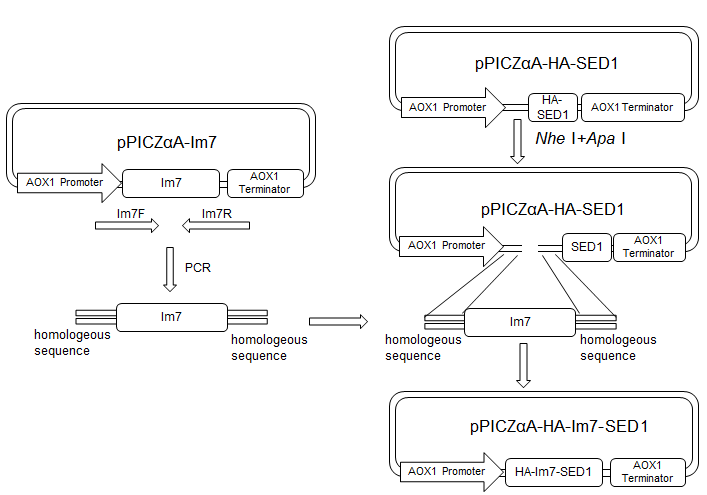


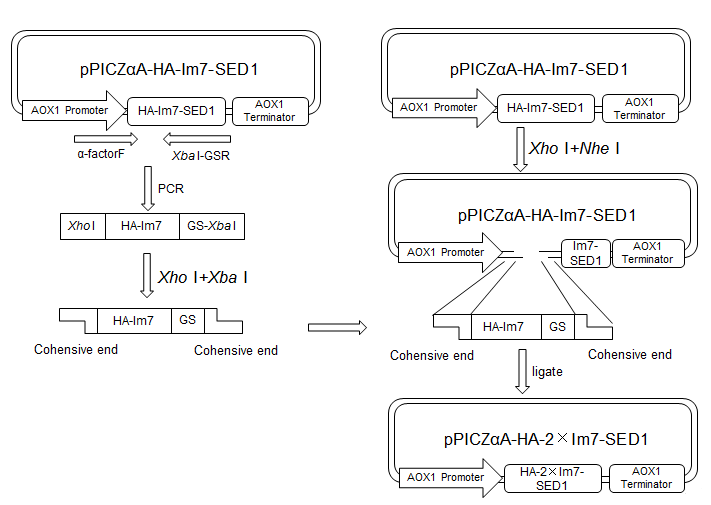


**Fig S3.** Scheme for the construction of plasmid pPICZαA-HA-2 x Im7-SED1.


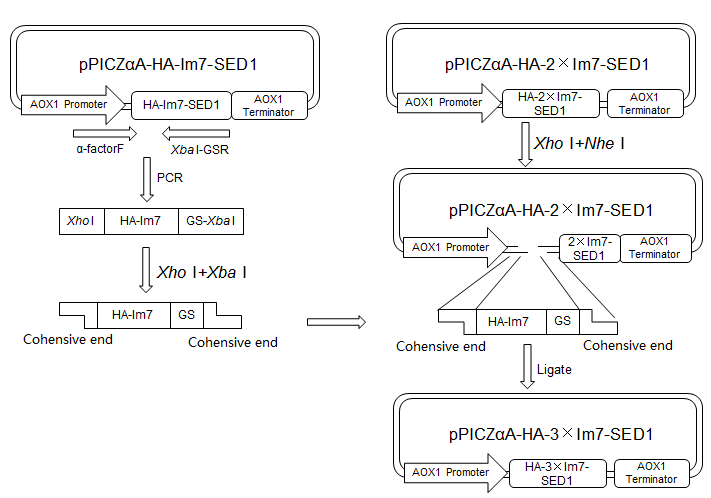


**Fig S4.** Scheme for the construction of plasmid pPICZαA-HA-3 x Im7-SED1.

**Table S1.** The primers used for the construction of *P. Pastoris* plasmids.

| **Primer** | **Sequence 5’ 3’** |
| --- | --- |
| SED1F | aggcggtagcggaggcggagggtcgcaattttccaacagtacatctgcttcttcc |
| SED1R | gaaagctggcggccgccgcggtcattataagaataacatagcaacaccagccaaac |
| ZαHAF | aaagagaggctgaagctgaattctacccatacgacgttccagactacgctggaggctct |
| HA-MCSF | cgttccagactacgctggaggctctgctagccatatggttaacgggcc |
| MCS-GSF | tgctagccatatggttaacgggcccggaggcggtagcggaggcggagggtc |
| Im7F | ctacgctggaggctctgctagcatggaattgaagaactccatctccgact |
| Im7R | ctccgctaccgcctccgggcccaccttgtttaaaacctggcttaccgttg |
| α-factor F | tgctaaagaagaaggggtatctctcgagaa |
| XbaI-GSR | ctgacttgcatctagacgaccctccgcctccgctaccgcc |


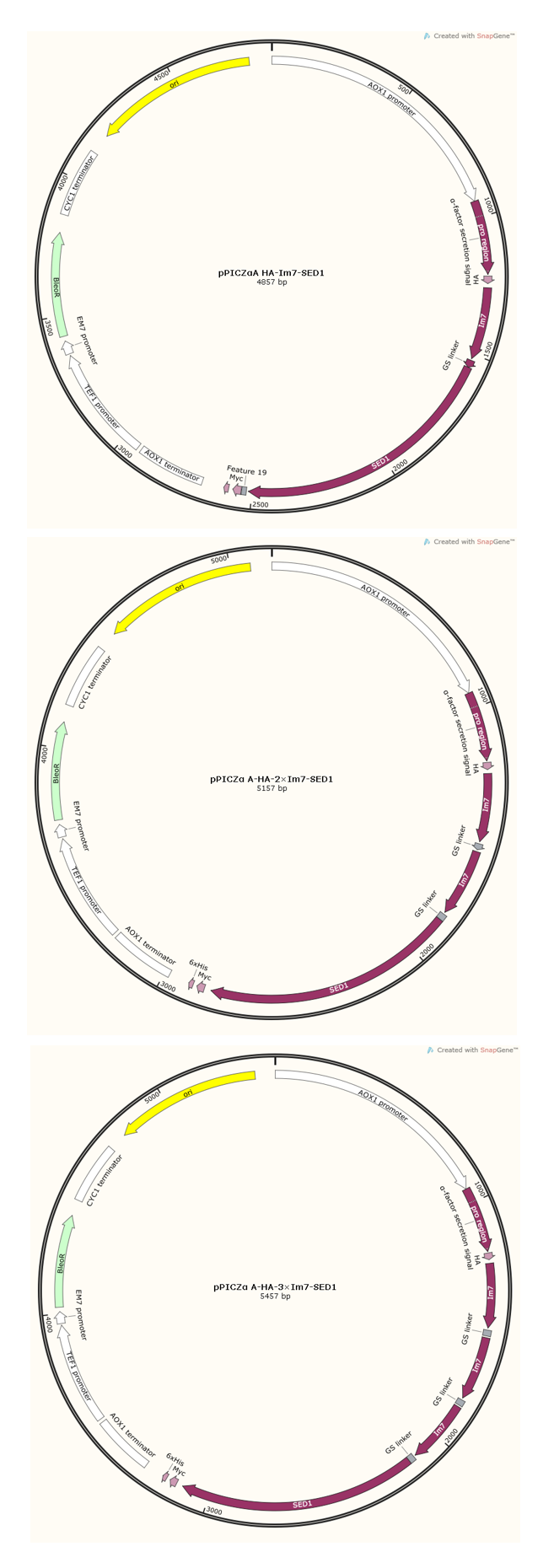


**Fig S5.** The illustrated maps of IM7 yeast cell surface displaying plasmids.





**Fig S6.** Enzyme activity of minicellulosome against CMC for fresh or stored Y-IM2 and Y-IM3 strains.
